# The case for microbial intervention at weaning

**DOI:** 10.1080/19490976.2024.2414798

**Published:** 2024-10-28

**Authors:** Julia N. Flores, Jean-Bernard Lubin, Michael A. Silverman

**Affiliations:** a Division of Infectious Disease, Department of Pediatrics, The Children’s Hospital of Philadelphia, Philadelphia, PA, USA; b Department of Microbiology, Perelman School of Medicine, University of Pennsylvania, Philadelphia, PA, USA; c Institute for Immunology and Immune Health (I3H), Perelman School of Medicine, University of Pennsylvania, Philadelphia, PA, USA; d Department of Pediatrics, Perelman School of Medicine, University of Pennsylvania, Philadelphia, PA, USA

**Keywords:** Microbiome, weaning, complementary feeding, immune system development, microbial ecology, probiotics

## Abstract

Weaning, the transition from a milk-based diet to solid food, coincides with the most significant shift in gut microbiome composition in the lifetime of most mammals. Notably, this period also marks a “window of opportunity” where key components of the immune system develop, and host-microbe interactions shape long-term immune homeostasis thereby influencing the risk of autoimmune and inflammatory diseases. This review provides a comprehensive analysis of the changes in nutrition, microbiota, and host physiology that occur during weaning. We explore how these weaning-associated processes differ across species, lifestyles, and regions of the intestine. Using prinicples of microbial ecology, we propose that the weaning transition is an optimal period for microbiome-targeted therapeutic interventions. Additionally, we suggest that replicating features of the weaning microbiome in adults could promote the successful engraftment of probiotics. Finally, we highlight key research areas that could deepen our understanding of the complex relationships between diet, commensal microbes, and the host, informing the development of more effective microbial therapies.

## Introduction

Trillions of microbes live on and inside the human body, reaching densities as high as 10^11^ bacteria/g in the large intestine.^1^ This collection of microbes, termed the microbiota, performs critical functions for the host across immunity, metabolism, and nervous system regulation. Thus, it is not surprising that disruptions in microbiota composition, often called dysbiosis, are linked to an ever-growing list of diseases.^2–5^ Given its significant and broad impact on human health, there have been efforts to modulate the microbiome to treat and prevent disease dating back hundreds of years. In the 4^th^ century, Ge Hong, a doctor of Chinese traditional medicine, described the use of human fecal slurry, called “yellow soup”, to treat severe diarrhea, among other conditions.^6^ In 1917, the first single organism probiotic, a commensal strain of *Escherichia coli*, was isolated from the feces of a soldier who was resistant to dysentery.^7^ Despite the longstanding use of live microbial therapeutics, only two drugs in this category have been approved by the Food and Drug Administration.^8^ One of the major challenges to developing successful microbial therapeutics is engraftment into the preexisting dense adult community. In this review, we propose that leveraging the principles of microbiome assembly and ecology in the context of early-life changes in diet, environment, and host physiology will support the development of successful microbial therapeutics.

Weaning is the most dramatic dietary “intervention” in the lifetime of most mammals. This transition from milk-based nutrition to a diverse and variable diet of plant and animal products profoundly changes the intestinal microbiome.^9^ An increase in microbial diversity and an orchestrated succession of changes to the composition of the intestinal microbiome at weaning are hallmarks of the transition toward a mature microbiome. The influx of new microbial taxa at weaning provides critical stimulation to the developing immune system, and disruption of host-commensal interactions in early life can negatively impact long-term immune homeostasis.^10–12^ Thus, we propose weaning as an ideal time to leverage microbial therapeutics to modulate the early life microbiome and promote healthy immune development.

## Nutritional transition at weaning and weaning practices

Breastmilk is widely regarded as the gold standard for infant nutrition because it supplies all the essential macronutrients and most of the micronutrients required for healthy development.^13,14^ While its composition is relatively consistent across human populations, breastmilk can vary depending on the infant’s age, between mothers, and even within individual feeds.^15–17^ Carbohydrates, mainly lactose and milk oligosaccharides, are the most abundant macronutrients in breastmilk.^18,19^ Lactose, a disaccharide, provides readily available energy for the growing infant.^20^ The concentration of lactose in human milk is considerably higher than in other species, and it is hypothesized that this abundance supports rapid brain development in human infants.^21^ Most lactose is absorbed in the small intestine, primarily feeding the infant.^20^

In contrast, milk oligosaccharides are complex carbohydrates that are largely indigestible by the infant. As such, they reach high concentrations in the lower gastrointestinal tract where they promote the growth of beneficial commensal bacteria.^22–25^ Human milk oligosaccharides (HMOs) are remarkably diverse with hundreds of unique structures, while the milk oligosaccharides of other mammals tend to be less varied.^26^ HMOs are also present at higher concentrations than oligosaccharides found in other mammals’ milk, suggesting that HMOs selecting for beneficial microbes may play a particularly important role in human development.

Fat is the second most abundant macronutrient in breastmilk. Milk fat is composed primarily of triglycerides (~98%), with the remaining fraction consisting of phospholipids, sphingolipids, and cholesterols. Almost all milk fat is packaged into milk fat globules, complex structures consisting of a tri-layer membrane of polar lipids and proteins surrounding a triglyceride core.^27^ Fat provides almost half of the energy content in breastmilk, as well as essential support for central nervous system development.^19,28,29^ Milk fat content can vary widely between species, rising as high as over 50% in pinnipeds and whales.^30^ The lipid composition of milk fat also varies by species. For example, while overall lipid content is similar in human (~3.7%) and cow (~3.3%) milk, human milk contains substantially higher proportions of polyunsaturated fatty acids and a higher ratio of omega-6 to omega-3 fatty acids.^21^

Protein is the third most abundant macronutrient in human breastmilk. Milk proteins are divided into two categories, whey and casein. Whey is soluble and is more easily digested, while casein forms difficult-to-digest solid curds.^31,32^ Human milk is low in overall protein content and has a much higher whey-to-casein ratio compared to other mammalian milk.^21,33^ Lower protein content in milk correlates to longer maturation time of offspring.^33^ In addition to protein, there are high concentrations of free amino acids in breastmilk, primarily glutamine and glutamate.^34,35^ Metabolism of free amino acids, urea, and other non-protein sources of nitrogen are implicated in the development of a healthy infant gut microbiome.^36^ Free amino acids also act as signaling molecules that shape the developmental trajectory of the infant, in concert with hormones, growth factors, and microRNAs (miRNAs) found in breastmilk.^37,38^

Maternal immune factors are arguably the most important non-nutritive component of breastmilk. These include lymphocytes, cytokines, chemokines, antimicrobial enzymes, and immunoglobulins, primarily secretory IgA (sIgA).^39^ These maternal immune factors, particularly maternal antibodies, provide passive protection against neonatal infections and promote healthy microbiome and immune maturation.^39–42^ Overall, lactation has evolved over tens of millions of years to provide ideal nutrition, developmental signals, and immunity to neonates.^43^

Infant formula is a manufactured liquid total nutrition meant to supplement or fully substitute for breastmilk. Most formulas are derived from cow milk, although there are soy-based and specialty elemental formulas for infants with food sensitivities, allergies, or other digestive and metabolic needs.^13^ As detailed above, the macronutrient composition of cow milk is different from that of humans, so it is modified to suit the needs of human infants as defined by strict national guidelines.^44,45^ Still, formula is not identical to human breastmilk. For example, cow milk-based formulas have a higher casein-to-whey ratio, different vitamin and mineral bioavailability, and different fat globule composition, structure, and size than human breastmilk.^46^ While breastmilk adapts to meet the changing developmental needs of the infant, infant formula has a constant composition. Importantly, formula does not contain the nonnutritive bioactive components found in breastmilk, including immune factors such as IgA. Additionally, the concentration and composition of milk oligosaccharides in bovine-based formula differ from human breastmilk.^26,40^ In light of their important role in promoting the growth of beneficial microbes, there is ongoing research on the effects of supplementing formula with HMOs.^47,48^

For the purposes of this review, “weaning” refers to the complementary feeding period from the first introduction of solid food to the cessation of breastfeeding or formula feeding. The World Health Organization (WHO) recommends exclusively breastfeeding until the introduction of first complementary foods at six months of age and continuing to breastfeed until at least two years of age.^49^ Globally, rates of exclusive breastfeeding in the first six months of life are low, ranging from 45% in low- and middle-income countries (LMICs)^50^ to less than 25% in the United States.^51^ In high-income countries, weaning typically starts and ends earlier than is recommended and consumption of infant formula is common.^52^ For example, in the United States, 20 to 40% of infants begin consuming solid food before four months of age,^53^ while only 35% still breastfeed at 12 months.^51^ In LMICs, infants more often breastfeed until two years of age or later, as recommended, although the use of infant formula to supplement or fully replace breastmilk is becoming more common in this setting.^52^ Despite better compliance with WHO infant feeding guidelines in LMICs, the nutritional transition over weaning may still be disrupted due to higher rates of maternal malnutrition which can affect breastmilk composition^54^ and food insecurity, which results in lower frequency and quality of meals after introduction of complementary foods.^55^ These differences in feeding behaviors contribute to some of the variation in microbiome development seen across geography and lifestyles, as will be discussed in more detail later in this review.

Although complementary feeding practices vary by culture, there are conserved nutritional differences between total milk-based nutrition and a solid food diet. In most cultural contexts, early complementary foods are grain-based products, like cereal and porridge, as well as fruits, vegetables, and breastmilk or formula.^56,57^ Foods that are part of the normal household diet, such as meat, yogurt, and cheese, sometimes called “family foods,” are introduced slowly over the first few years of life which gradually results in a more diverse, adult-like diet. Early in the complementary feeding period, the overall macronutrient balance and energy intake remain relatively stable because infants typically consume less milk in proportion to the addition of solid food to their diet.^19,58^ In other words, the nutritional content of early complementary foods replaces nutrients that would otherwise have come from breastmilk or formula.^19,58^ As infants develop, they begin to consume more solid food to meet their growing caloric and nutritional needs.^58^ During this time, the proportion of energy provided by carbohydrates increases while the contribution of fat gradually decreases from about 50% of total calories to 20–30%.^19,58^ Early complementary foods, like cereals, are lower in micronutrient concentration than breastmilk or formula, so they are often fortified with iron, zinc, and other vitamins and minerals.^58^ The most notable nutritional change at weaning is the introduction of complex polysaccharides not found in breastmilk or formula, including dietary fiber and animal glycans.^59^ Like milk oligosaccharides, dietary fiber is resistant to digestion and is an important source of microbially available carbohydrates.^60^ While breastmilk is rich in poly-unsaturated fatty acids, complementary foods are often low in these essential lipids and can be high in saturated fat and cholesterol.^58^ The proteins found in dairy products are similar to the whey and casein found in breastmilk, but meat, legumes, and vegetables are sources of novel proteins.^58^ Complementary foods vary across cultures, lifestyles, and individuals,^57^ but they generally increase the diversity of available nutrients. Solid foods also introduce different textures and densities of material to the diet that subsequently change stool consistency and provide critical stimulation to the developing enteric nervous system.^61^ Lastly, the frequency of feeding decreases over weaning from nursing every few hours to three solid food meals per day.^56^ This change in meal frequency may impact the microbiome at weaning since microbial composition and metabolism exhibit diurnal fluctuations in adults associated with the timing of meals.^62^ These dramatic changes in nutrient composition, food texture, and feeding frequency lead to an equally dramatic shift in the microbes that stably colonize the gut during and after weaning.

## Microbiome composition and dynamics during weaning

Early microbiome development, including the seeding of first commensal microbes and the first stages of community assembly, has been reviewed extensively.^63–65^ In brief, humans are sterile *in utero* and, under healthy conditions, are first exposed to live microbes when amniotic membranes rupture during labor or Cesarean section (C-section).^66,67^ Signatures of birth mode can be detected in the first days to weeks of life in the intestinal microbiome, with a subset of species of the vaginal microbiome colonizing vaginally delivered infants, and a subset of skin-associated microbes colonizing those delivered by C-section.^68,69^

Despite differences in initial colonization, the human microbiome is remarkably well-conserved across geography, lifestyles, and study designs.^70–72^ Alpha diversity, the diversity within a sample, is relatively low in the infant gut until weaning when richness and evenness increase dramatically, eventually reaching near-adult levels by three to five years of age.^9,71,73,74^ Similarly, the absolute abundance of intestinal microbes increases exponentially over weaning from ~ 10^8^ bacteria/g in infants to 10^11^ bacteria/g in adults.^1,75^ In contrast to alpha diversity, beta diversity, the distance between samples or groups, decreases over weaning. As children age, their microbiomes exhibit smaller compositional changes over time and converge to more closely resemble the microbiomes of children of the same age.^71,73,76–78^ These processes result in microbial communities that become increasingly similar to healthy adult samples.

### Pre-weaning microbiome

#### Humans

Members of the phylum *Pseudomonadota* (previously *Proteobacteria*), represented mainly by microbes from the family *Enterobacteriaceae*, are some of the earliest colonizers of the human gut. They reach a peak relative abundance of ~ 20 to 40% within three months of life and then decrease in abundance until stabilizing at less than 5% after weaning.^68,79,80^ This rapid decline in the relative abundance of *Enterobacteriaceae* starts before the introduction of solid food, suggesting that it is not driven by dietary changes. It is not well understood why *Enterobacteriaceae*, including microbes with pathogenic potential, bloom early in development. The prevailing theory is that they are facultative anaerobes that tolerate and thrive in an oxygen-rich environment.^81^ It has been proposed that these microbes deplete oxygen in the gut lumen which allows for later colonization by anaerobes that subsequently outcompete them. Yet germ-free and conventional mice have similar concentrations of oxygen in their intestinal lumen, which calls into question whether oxygen consumption by microbes can fully explain the transition from facultative anaerobes to strict anaerobes.^82^ Additionally, *Enterobacteriaceae* that colonize the meconium immediately after birth produce metabolites consistent with anaerobic fermentation, also raising questions as to whether early-life community dynamics are driven primarily by the redox state of the gut.^83^ Another possible explanation, that we favor, is that the broad metabolic capabilities and faster growth rates of *Pseudomonadota* give these taxa an advantage during early colonization, but over time, they fail to compete with slower-growing milk specialists.^80,82,84^

Microbes that thrive on a milk-based diet include lactic acid bacteria of the phylum *Bacillota* (previously *Firmicutes*), namely *Lactobacillus, Staphylococcus*, *Streptococcus*, and *Enterococcus* species. *Lactobacillus* species, as the name suggests, efficiently utilize lactose to produce lactic acid and have been used for centuries to make dairy products like cheese and yogurt.^85,86^ Members of the genus *Veillonella* consume the lactic acid produced by lactic acid bacteria and therefore also bloom on a milk-based diet.^87^ These early-life associated *Bacillota* typically reach a peak relative abundance of about 10% at 2 to 6 months of age and, unlike *Enterobacteriaceae*, remain abundant until the cessation of breastfeeding.^3,70,77,87^ Members of these genera have been detected in expressed breastmilk, but there is controversy surrounding their origin.^88^ Studies in mouse models and in humans given oral probiotics suggest that maternal intestinal microbes may traffic from the intestine and subsequently seed the infant’s intestine via consumption of breastmilk.^89,90^ In humans, *Staphylococcus*, *Veillonella*, and *Streptococcus* are also dominant members of the skin and oral microbiome.^91–93^ As such, members of the “breastmilk microbiome” may originate from breast skin and/or retrograde flow from the infant’s oral cavity before colonizing the gut.^94,95^ *Lactobacillus* species are highly abundant in the vaginal microbiome, but studies of mother-infant dyads reveal that the species and strains seen in maternal vaginal samples do not persist in neonatal stool past the first few days of life and are quickly replaced by gut-associated strains.^69^ The question of how neonates become colonized with intestinal maternal lactobacilli and other early life-associated *Bacillota* remains an active area of research.

While early-life-associated members of *Bacillota* compete with the host to utilize the simple carbohydrates in breastmilk, members of *Actinomycetota* (previously *Actinobacteria*), represented almost entirely by *Bifidobacterium*, encode extensive machinery to break down HMOs, giving them access to a carbon source not utilized by the host.^96,97^ Thus, it is not surprising that *Bifidobacterium* species are highly abundant and prevalent in breastfeeding infants as compared to formula-fed infants,^71,79,98^ reaching relative abundances greater than 50% in some populations.^3,77,78,87^ *B. longum* subsp. *infantis* is the most efficient HMO utilizer, but *B. longum* subsp. *longum, B. bifidum* and *B. breve* can also metabolize HMOs.^99–103^ Multiple *Bifidobacterium* strains can colonize a single individual, forming a cross-feeding network that allows for the efficient breakdown of hundreds of different HMO structures.^104–106^ The sugar subunits cleaved off during HMO metabolism can then be utilized by other commensals that do not encode these specialized enzymes.^104^ Infants with lower levels of *Bifidobacterium* tend to have a higher abundance of *Bacteroides* species.^79,107^ *Bacteroides* metabolize a broad range of complex carbohydrates, including HMOs, allowing them to occupy that metabolic niche.^108^ *Akkermansia muciniphila* and *Clostridiales*, including *Roseburia* species, also metabolize HMOs and are prevalent in breastfeeding infants.^23,73,107,109^ Interestingly, HMOs are structurally similar to mucus glycans, so bacteria typically considered mucolytic, like *A. muciniphila* and *Bacteroides thetaiotaomicron*, can use the same set of enzymes to bind and cleave off the carbohydrate moieties from both mucus and HMOs.^25,59,108,110,111^ Similarly, *Bifidobacterium* species can grow on mucus glycans.^112^ It has been proposed that HMOs help select for commensal microbes that can metabolize a wide array of carbohydrates, including mucus glycans. This metabolic diversity allows HMO specialists to engraft in the intestinal mucus layer by providing them with a competitive advantage during weaning and promoting strain transfer from mother to infant.^110,111,113–115^ Altogether, the metabolism of complex carbohydrates is an important driver of colonization dynamics over the weaning period.

#### Non-human mammals

The pre-weaning microbiomes of humans and other monogastric mammals are similar in many ways. Pre-weaning rodents in laboratory settings are dominated by *Enterobacteriaceae* and *Lactobacillaceae*, transitioning to a community dominated by *Clostridia* and *Bacteroidota* in adulthood.^116,117^ Larger mammals, such as dogs and pigs, also have increased *Enterobacteriaceae* and *Clostridiaceae* colonization before weaning.^118–121^ The *Clostridiaceae* family includes several pathogenic species such as *Clostridium botulinum, C. tetani* and *C. difficile*. Remarkably, human infants are often asymptomatically colonized with toxigenic *C. difficile*.^122^ Similarly, Guard et al.^123^ found significant levels of *Clostridium perfringens*, a disease-causing agent in adult dogs, colonizing neonatal puppies. These findings suggest that pathogenic *Clostridiaceae* species often colonize mammalian neonates without causing disease. Pre-weaning non-human primates also have higher proportions of *Enterobacteriaceae*, *Enterococcaceae, Veillonella, Akkermansia* and *Streptococcus* species.^124,125^ Overall, pre-weaning microbiomes are well conserved across mammals, with the most abundant bacterial clades (e.g., *Bacteroidaceae, Bacilli, Enterobacteriaceae, Clostridiaceae*) represented in all species we surveyed.

There are notable exceptions to the commonalities among pre-weaning microbiomes that are important to consider especially when using different model organisms to study the early-life development of the microbiome. *Bifidobacterium* species dominate the microbiomes of breast-fed human infants yet are minor constituents of the pre-weaning microbiome in non-human primates. *Bifidobacterium* is also conspicuously absent in the more evolutionarily distant mammalian species (rodents, carnivores, and pigs) surveyed for this review. Instead, *Bacteroidaceae* are the dominant colonizers of the pre-weaning gut in non-human primates, rats and pigs. *Bacteroidaceae* are the primary consumers of milk oligosaccharides in pre-weaning piglets,^120^ likely replacing the functions of *Bifidobacterium* in these species. The dominance of the *Bacteroidaceae* in non-human mammals, suggests that human infant-associated *Bifidobacterium* have evolved specifically within the context of the human neonatal gut environment, and this close relationship should be considered when interpreting bifidobacterial function in animal models.

### What drives the weaning microbiome transition?

#### Humans

**Figure 1.**
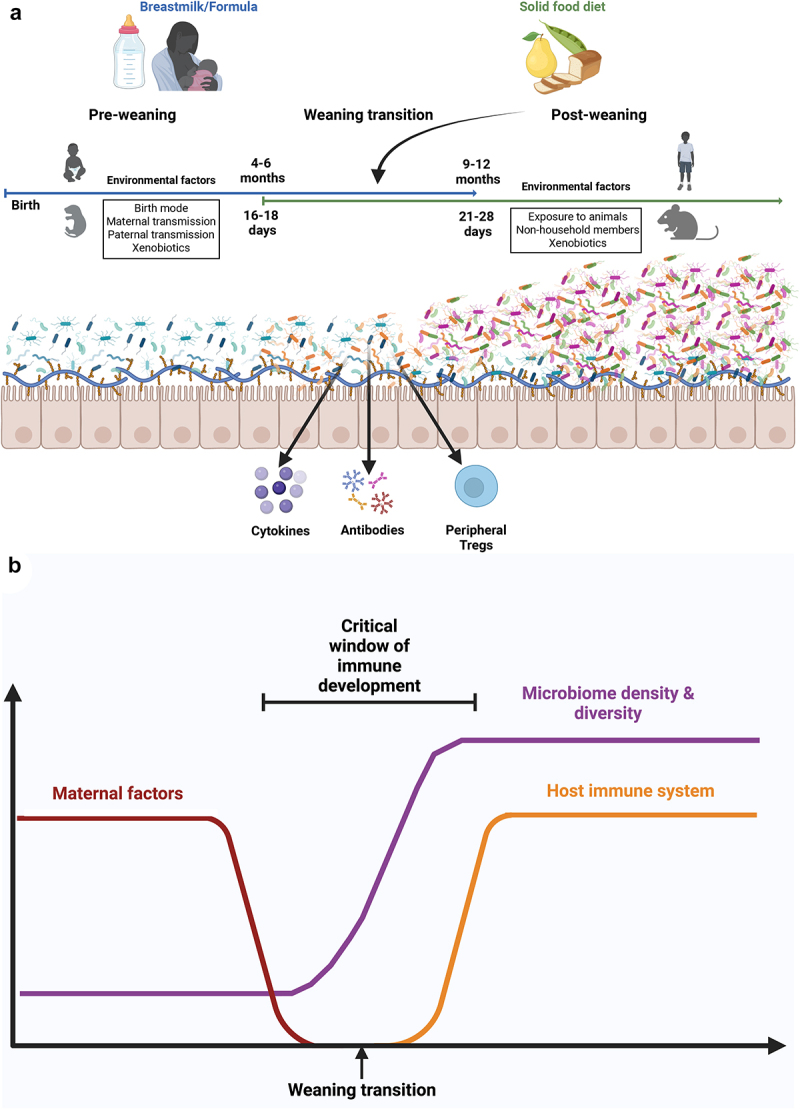
Weaning-associated microbiome and immune system development.

The combined effects of the introduction of solid food and the cessation of breastfeeding shape the changes in microbiome diversity and composition during weaning (Figure 1). The marked increase in alpha diversity at weaning is primarily attributed to the introduction of solid food,^65,87,126^ as many of the newly abundant taxa have the capacity to break down complex carbohydrates, including plant polysaccharides and animal glycans. However, the dominance of *Bifidobacterium* in the human gut during breastfeeding can result in especially low alpha diversity pre-weaning and a subsequent increase in diversity can occur after the cessation of breastfeeding, independent of the introduction of solid food.^3,70,77,79^ Cessation of breastfeeding is also associated with a decrease in the abundance of early-life *Bacillota*, such as *Staphylococcus*, *Streptococcus*, *Veillonella*, and *Lactobacillus*, as well as *Bifidobacterium* species.^3,70,77,87^ Overall, the cessation of breastmilk and introduction of solid food drive specific changes in microbiome features that together account for the well-conserved developmental trajectory of the microbiome during weaning.

a) Model showing that the dietary transition from milk to solid food at weaning leads to significant changes in the composition, density, and diversity of the intestinal microbiota. This dramatic shift in the microbiome corresponds with major milestones in immune system development, such as the induction of peripheral regulatory T cells, endogenous sIgA, and cytokines as part of the “weaning reaction”.^11^ b) Schematic showing the loss of maternal factors and increase in microbiome density and diversity at weaning marks a window of opportunity in which host-microbe interactions induce tolerogenic immune responses that impact long-term immune homeostasis.

Many of the taxa that bloom upon the introduction of solid food belong to the “fiber-loving” class *Clostridia*, including *Blautia*, *Ruminococcus*, and *Faecalibacterium*.^60,74,87,98^ Other abundant taxa include species and strains of *Bifidobacterium* and *Bacteroides* that are closely related to those that grow well on HMOs, but instead encode enzymes that are more adept at degrading plant polysaccharides and animal glycans.^78,127,128^ For example, while *B. infantis* is strongly associated with the pre-weaning microbiome, *B. longum* persists and expands to relatively high abundance in adulthood.^80,129^ This transition reflects the capacity of *B. longum* to efficiently metabolize plant fiber.^127^ In their ecological model of microbiome assembly, Coyte et al.^130^ demonstrated that a change in available resources, such as the dietary transition at weaning, requires the existence of a transitional sub-community that can exist in either condition. Indeed, a recent longitudinal study following infants in rural Bangladesh, where weaning spans six months to two years of age, found a unique “transitional” *Bifidobacterium* strain that expresses machinery to metabolize both HMOs and plant polysaccharides and blooms during mixed feeding.^131^ Another recent study of a Finnish cohort of healthy mother-infant dyads conducted deep metagenomic sequencing and found genes with 100% nucleotide identity between mother and infant samples that are present in distinct genera and even phyla, indicating horizontal gene transfer.^132^ In addition to predicted mobile genetic elements, these shared sequences encoded genes predicted to be involved in carbohydrate and amino acid metabolism and iron acquisition, indicating that these genes may confer infant-associated microbes with adult-associated metabolic functions to aid in nutritional transition at weaning.^132^ These examples provide evidence that a broad metabolic capacity is necessary for microbes to bridge the transition of dietary substrates at weaning.

An important caveat to studies of the microbiome, and the early-life microbiome in particular, is that most studies rely upon relative abundance measurements that can be tricky to interpret in the setting of increasing microbial density in the gut. This approach can lead to spurious conclusions of taxa “crashing” when a decrease in relative abundance is actually due to a substantial increase in the absolute abundance of other taxa. For instance, in a study that measured both relative and absolute abundance in the developing microbiota of pre-term infants, *Staphylococcus* and *Enterococcus* became nearly undetectable after 40 days of life by relative abundance, despite the absolute abundance of each remaining fairly stable, indicating that these taxa may not have lost fitness but rather that other taxa had bloomed.^133^

Another important consideration in microbiome research is the significant role of cultural context and diet in shaping the post-weaning microbiome. The vast majority of microbiome research is conducted in high-income, Western countries where the adult diet is dominated by animal protein, refined grains, and calorically dense, processed foods.^134^ This differs from lower-income, less industrialized countries where staple foods are typically unrefined grains, vegetables, and plant protein in the form of legumes.^135^ Studies of hunter-gatherer and agropastoral communities from South America, Africa, and Asia reveal significant differences in microbiome composition compared to high-income industrialized Western populations. Adult members of these non-industrialized communities have markedly higher alpha diversity, increased abundance of *Prevotella* species, lower abundance of *Bacteroides* and *Bifidobacterium* species, and contain unique taxa, including *Treponema*, *Xylanibacter*, *Butyrivibrio*, and *Succinivibrio* species.^136–144^ These conserved differences contribute to an increased capacity of microbiomes from less industrialized populations to extract nutrients from complex plant polysaccharides like xylan, cellulose, and pectin.^145^

A subset of these studies focus on the microbiomes of infants and children from traditional communities to understand the developmental trajectory that leads to higher diversity and distinct compositional features in adults.^71,136,146,147^ A recent meta-analysis compared infant stool samples from the Hadza hunter-gatherers of Tanzania to 17 other publicly available microbiome datasets from children 0–36 months old living in industrialized, non-industrialized, and transitional communities.^72^ They found that microbiome composition is most similar across lifestyles in the first 6 months of life when all groups are dominated by *Bifidobacterium* and *Streptococcus* species.^72^ At 6 months, industrial populations diverge from non-industrial and transitional populations, exhibiting a bloom of *Bacteroides* and *Ruminococcus* species, likely reflecting the earlier introduction of solid foods.^72^ Samples from non-industrial and transitional communities do not diverge until about 30 months of age, when non-industrial communities contain more unique taxa not found in the other populations, mirroring what is seen in studies of adults.^72^ Microbial studies on paleofeces suggest that the microbiome composition of modern, non-industrialized populations more closely resembles that of prehistoric humans.^148,149^ It is posited that these relatively recent microbiome changes found in industrialized populations may contribute to the rising incidence of inflammatory diseases.^150,151^

The loss of VANISH (Volatile and/or Negatively associated in Industrialized Societies of Humans) taxa and increased prevalence of BloSSOM (Bloom or Selected in Societies of Urbanization/Modernization) taxa in industrialized populations highlight the need to understand how we acquire our commensal microbes.^152^ Shotgun metagenomic sequencing of microbiome samples provides strain-level resolution which allows for more accurate inference of transmission patterns than the higher order taxonomic classification provided by 16S rRNA gene sequencing.^153^ In studies of microbiome development, most strain-level analyses have focused on the vertical transmission of microbial taxa from mothers to infants at early time points. Within hours to days after birth, an infant’s microbiome is relatively homogeneous across barrier sites and the composition roughly reflects the maternal body surface encountered during birth, i.e., the vaginal microbiome for vaginally birthed babies and the skin microbiome for babies delivered by C-section.^69,154^ During the first few months of life, the strains shared between mother and infant are mainly members of the maternal gut microbiome, with *Bacteroides* and *Bifidobacterium* among the commonly shared taxa.^69,155,156^ Infants also share strains with other household members regardless of genetic relatedness, pets, and even hospital surfaces.^157–160^ A recent study reported the rate of strain sharing between fathers and their infants increases over time, matching the rate of maternal strain sharing by one year of age.^161^ They also found that these paternal strains do not overlap with shared maternal strains, indicating that each parent provides a unique contribution to their child’s microbiome.^161^

Fewer metagenomic studies have focused on later time points that include the apparent acquisition and bloom of specific taxa at weaning. A recent meta-analysis of pooled metagenomic datasets from 20 populations spanning geography and lifestyles that included data on familial relationships and cohabitation revealed that strain sharing follows a “social distance-based gradient,” with the highest rates of strain sharing between cohabitating mothers and their children 0–3 years old, followed by other household members, and lastly non-cohabitating adults in the same community.^156^ Over time, children share fewer strains with their mother and other household members and are colonized by an increasing proportion of unique strains that come from other environmental sources.^156^ These findings support a model of continuous seeding of new microbial strains from the environment, whether that be through direct physical contact with other individuals or a shared environment, that leads to shifts in the dominant strain of a given species over time.

Interestingly, Valles-Colomer et al.^156^ found similar rates of strain sharing across populations, despite different geographic locations and lifestyles. They observed that the likelihood of transmission of a given strain was not associated with its prevalence or relative abundance in a population but rather correlated with microbe-specific features. For example, Gram-negative *Proteobacteria*, which are generally more resistant to disinfectants,^162^ exhibited a higher frequency of maternal and household transmission, while transmission between non-cohabitating members of the same community occurred more frequently among aerotolerant and spore-forming species.^156^ These data underscore the need to consider the impact of modern sanitation, hygiene practices, and antibiotics usage on the strains that are circulating among human populations and make efforts to avoid the loss of beneficial taxa.^163^ In this vein, there are ongoing projects to catalog and preserve the microbial diversity found in more traditional communities in open-access biobanks.^164^

#### Non-human mammals

In other mammalian species, major changes also appear in the most abundant bacterial lineages at weaning. Like in humans, the weaning period in rodents, carnivores, pigs and non-human primates is accompanied by increasing gut microbiome alpha diversity.^120,125,165^ This increase in bacterial diversity is characterized by the colonization of microbes primarily from the *Lachnospiraceae* and *Ruminococcaceae* families of *Clostridia* and *Bacteroidota* and reduction of *Pseudomonadota*, mirroring the taxonomical shifts that occur in humans. *Verrucomicrobiota* consistently decreases during weaning across mammalian species,^117,125,166,167^ providing evidence of *Akkermansia* as an early-life associated microbe. In carnivorous mammals such as dogs and cats, weaning leads to a substantial increase in *Fusobacterium* colonization.^119,168^ Similarly, *Fusobacterium* colonization in humans is positively associated with a high-fat and protein diet, further linking *Fusobacterium* to meat consumption.^169^

The families of *Bacteroidota* that dominate post-weaning animals tend to differ from humans. The family *Muribaculaceae* (formally annotated as S24–7) often dominates the gut of mice and other rodents post-weaning at the expense of *Bacteroidaceae* species.^12,170,171^ High abundances of *Prevotellaceae* are more typically found in the post-weaning microbiome of pigs^120,121^ and non-human primates.^124,125^ The increased prevalence of *Prevotella* over *Bacteroides* species in post-weaning pigs and non-human primates is thought to be an indicator of increased fiber intake of these species and degradation capabilities of these microbes. In Western populations, *Bacteroides* is the most abundant *Bacteroidota* genus after weaning and is correlated with decreased fiber intake relative to non-Western populations. *Prevotella* dominance in non-Western populations and ancestral human post-weaning microbiomes likely reflects common features of these groups’ diets. Overall, diet is a principal driver of the diverging microbiomes of humans and other mammals at weaning, with the amount of meat and fiber consumption being the greatest contributing factors.

The weaning transition in piglets has been extensively studied because they can develop weaning stress illness as a result of early and abrupt weaning practices in commercial farming.^172^ Weaning stress is generated by several deleterious morphological and immunological changes including increased villi shedding and mucosal atrophy, disruption of tight junction formation between epithelial cells, mast cell hyperplasia and increased degranulation, and increased levels of pro-inflammatory cytokines (TNF-α, IFN-γ, IL-1β, IL-6 and IL-8).^172^ These alterations trigger decreased epithelial barrier function, reduction in digestive enzyme secretion and diarrhea, that ultimately lead to malnutrition and reduced growth in affected animals. Studies in pigs found a decrease in alpha diversity in the immediate days that follow abrupt weaning, coinciding with increased colonization of potentially pathogenic *Campylobacter* species.^166^ Weaning stress in piglets suggests that a transitionary period of mixed milk and solid food feeding is important for healthy early-life development.

### Non-bacterial microbiota during weaning

While most studies on microbiome development focus on the bacterial microbiome, more recent studies have highlighted the contribution of viruses, fungi, and eukaryotic microbes. The dynamics of these organisms within and across microbial kingdoms likely contribute to the development of a healthy microbiome and host.

#### Virome

The intestinal virome is dominated by bacteriophages that begin to colonize in the first weeks of life.^173,174^ The bacteriophages detected at these early time points are induced from the earliest bacterial colonizers of the gut.^75^ The earliest comprehensive assessment of the infant intestinal virome reported that bacteriophage diversity was greatest in the first week of life,^175^ however, more recent studies have found increasing diversity during the weaning period.^75,173,176^ This discrepancy is likely due to better classification of viral genomic signatures in metagenomic sequencing samples. Bacteriophage content is likely altered by weaning through the changes in bacterial composition associated with solid food intake, e.g., increases in bacteriophages that infect *Bacteroidota* and *Lachnospiraceae* post-weaning.^173^

Unlike the bacteriophage component of the intestinal virome, eukaryotic viruses are typically undetectable before three months of age. The most prevalent families of the early-life eukaryotic virome are associated with neonatal infections including: *Picornaviridae* (Enterovirus, Rhinovirus), *Caliciviridae* (Norovirus, Sapporovirus), *Reoviridae* (Rotavirus), as well as *Circoviridae*, *Anelloviridiae*.^75,175,177^ Viruses of the family *Anelloviridiae* are correlated with age, with a spike occurring during the weaning period.^175,177^ It is speculated that maternal IgG in breastmilk inhibits viral colonization before weaning, leading to an influx of novel viruses at the start of solid food intake.^75^ Anelloviruses are found in ~ 90% of healthy adults without any associated pathologies.^178^ How these potential ‘commensal’ viruses interact with the immune system, particularly during the weaning transition, is unknown. Additionally, despite weaning-associated shifts in the virome, the absolute abundance of viruses peaks at 1 month of age and remains stable into adulthood.^75^ This raises the question of why the absolute abundance of viruses does not increase proportionally to the abundance of microbes in the gut. Further studies are needed to better understand the role that viruses play in shaping microbial colonization and immune system development during weaning.

#### Mycobiome

The intestinal fungal community, or mycobiome, also undergoes weaning-associated changes. Infants are colonized by fungi including *Candida*, *Malassezia*, and *Debaryomyces* species.^179–183^ A detectable maternal mycobiome increases the likelihood of fungal colonization in offspring,^180^ suggesting that vertical transfer is the primary source of commensal fungi in early life. Apart from the first two weeks of life, mycobiome alpha diversity is either unchanged or decreased from pre- to post-weaning timepoints.^182–185^ Likewise, mycobiome alpha diversity in pigs remains stable^186^ or decreases^187^ from birth to weaning. This contrasts with the development of the bacterial microbiome, where an increase in alpha diversity post-weaning is a hallmark of bacterial microbiome maturation. However, there are compositional differences between pre- and post-weaning mycobiomes,^184,188^ as well as an overall increase in fungal load.^183^ Early-life infant mycobiomes contain higher abundances of molds, particularly of the genus *Penicillium*, than post-weaning infants.^183,184^ Higher abundances of *Malassezia* and *Candida* spp. are also associated with pre-weaning mycobiomes.^182,184,188,189^ It was recently reported that a sub-population of human infants (20%) exhibited increased fungal alpha diversity through weaning.^185^ This ‘atypical’ population was characterized as having a higher abundance of *Candida* pre-weaning, relative to infants that followed the typical trajectory of decreasing fungal diversity. This high abundance of *Candida* persisted post-weaning at the expense of *Saccharomyces*, which typically increases in post-weaning fungal communities.^182,184,185^ Thus, there are clear differences in the gut mycobiome during ontogeny, most strongly associated with the ratio of *Candida* to *Saccharomyces* pre- and post-weaning.

It is disputed whether there is a stable, resident mycobiome in the gastrointestinal tract, further complicating our understanding of weaning-induced changes in the mycobiome. It has been argued that the bulk of intestinal mycobiota are transient species, derived from the environment, food sources or oral cavity fungal colonizers.^190,191^ This potentially explains the high inter- and intra-subject variability of fungal communities in the gut, though there are studies that argue for a persistent core mycobiome in the gut.^192,193^ Weaning-associated shifts in relative abundance have been observed among the core fungal colonizers *Saccharomyces cerevisiae, Malassezia restricta*, and *Candida albicans*. More robust studies are needed to distinguish between resident commensals and transient species in infant populations and to reliably assess the impact of weaning on the mycobiome.

Although the extent to which weaning-associated dietary changes shape the compositional differences in pre- and post-weaning mycobiomes is not well established, a few examples suggest that diet is associated with specific fungi. Pre-weaning mycobiomes have been associated with a predominance of *Debaryomyces hansenii*, ^180,181^ a fungus commonly associated with cheese.^194^ This association suggests that fungi predisposed to metabolizing milk components preferentially colonize the infant gut at pre-weaning ages. Additionally, the increase of *Saccharomyces* species during weaning has been associated with the introduction of yeast-containing products such as bread.^183,190^ However, the strongest predictors of mycobiome composition during ontogeny appear to be bacterial alpha and beta diversity.^185^ This highlights the importance of inter-kingdom interactions in the development of the intestinal microbiome. How the host is impacted by weaning-associated shifts in mycobiota is not well understood, though associations with body mass index and autoimmunity have been proposed.^183,189^ Further study is needed to clearly understand bacterial-fungal dynamics at weaning, the role that diet plays in these interactions, and the long-term consequences to host health.

## Weaning-associated microbiome dynamics vary across intestinal biogeography

The location of commensal microbes in the gut in the longitudinal and radial axis profoundly influences microbial interactions with the host.^195^ Microbiome composition, pH, mucus thickness, and resident immune cell populations vary along the longitudinal axis of the gastrointestinal tract while the concentration of oxygen and antimicrobial peptides, as well as unique microenvironments on food particles in the lumen and within the mucus layer, vary along the radial axis.^196^ Human microbiome studies typically use feces, as sample collection is easy and noninvasive, yet this limits analysis to the luminal contents of the distal intestine. Indeed, animal studies and more invasive human studies have described significantly different communities in the small intestine, colonic mucus layer, and intestinal crypts.^197,198^ The proximal small intestinal microbiome in adult mice is less diverse and contains a higher abundance of *Lactobacillus* species than the microbiomes found in the ileum and large intestine.^199^ The human small intestine is dominated by *Streptococcus*, *Veillonella*, and *Prevotella* species.^200^ These distinct communities reflect the unique environmental conditions in the small intestine.

The small intestinal microbiome is less impacted by the weaning transition than in the large intestinal microbiome, exhibiting smaller increases in alpha diversity and a composition more similar to the pre-weaning community.^118,170^ The small intestinal microbiota is likely buffered from weaning-associated changes due to the abundance of microbes that compete with the host for dietary lipids and simple sugars, which, unlike dietary fiber, are present in both pre-weaning and post-weaning diets. The limited compositional shifts in the small intestine at weaning are driven by increased production of bile acids which inhibit the growth of many bacterial species.^170^ When developing early-life microbial therapeutics, it is important to consider the unique weaning-associated dynamics affecting different biogeographical niches along the gastrointestinal tract.

There are fewer studies on the microbial communities that inhabit the intestinal mucosal surfaces, and the data that is available reports more variation in the mucosal microbiome as compared to the luminal community. This variation is likely due to differences in sample collection methods, which range from imaging fixed intestinal mucus to mucus scraping to homogenizing whole tissues.^201^ In mice, the mucus layer consists of microbes that metabolize complex carbohydrates, including *Lachnospiraceae*, *Muribaculaceae*, *Ruminococcaceae*, and *Clostridiales*. ^198,202,203^ Human mucosal samples, which include tissue biopsies and brush cytology samples, identified *Bacteroides*, *Streptococcus*, *Lachnospiraceae*, *Enterobacteriaceae*, and *Prevotella* species, among others.^201,204^ Improved methods for sampling human intestinal contents under physiological conditions are being developed, such as ingestible devices that sample as they pass through the intestinal tract.^205^ As far as we can find, there is no literature describing the changes in the mucosal microbiome over weaning. The changes in the intestinal microbiome at weaning should be considered as distinct processes occurring across multiple microenvironments and influenced by the different conditions found in each of these ecosystems. Considering the immunomodulatory properties of several mucus-associated microbes ^206–209^ (Table 1) and the immunologic maturation driven by microbial exposures at weaning,^10–12,212^ a more detailed understanding of microbial dynamics in the intestinal mucus during weaning will inform the development of early-life microbial interventions.

**Table 1.** Immunomodulatory commensal bacteria referenced in this review.

Bacterial taxa	Mechanism	Response	Reference
Escherichia coli Nissle 1917	Outer membrane vesicles	↑ IL-10, ↓ IL-1β, TNF-α, IL-17	^7^
Gram+ bacteria (Clostridia), Segmented Filamentous Bacteria (SFB)	Bacteria antigens, SCFAs	Weaning reaction (spike in TNFα, IFNγ)	^11^
*Peptostreptococcus russellii* DSM 23041	Indoleacrylic acid production	↑ globet cell differentiation, ↑ *Muc2* IL-10, ↓ IL-6, IL-1β	^206^
*Akkermansia muciniphila*	Amuc_RS03735 antigen	↑ T follicular helper cells	^207^
*Bacteroides fragilis* NCTC9343	Polysaccharide A	↑ Foxp3+ regulatory T cells, IL-10	^208^
*Bifidobacterium infantis* EVC001	Indole-3-lactic acid production	↑ Th1 cell polarization ↓ Th17 cell activation, ↑ IFNβ ↓ IL-13, IL-17A, IL-21, IL-31, IL-33, MIP3a	^210^
Probiotic consortium (SIMFORT® - VITAFOR) *Lactobacillus casei, Lactobacillus lactis, Lactobacillus acidophilus, Bifidobacterium bifidum, Bifidobacterium lactis*	Butyrate production	↑ Foxp3+ regulatory T cells	^211^

In summary, the microbiomes of humans of different populations are most similar during the pre-weaning period, and differences in diet and lifestyles lead to diverging communities during ontogeny. This is also the case when comparing human infants to other mammalian species. The occurrence of transitionary strains of bacteria during complementary feeding suggests the weaning period is distinct from the pre- and post-weaning environments. Coupled with the evidence that a lack of a transitional period leads to weaning stress and dysbiosis, the weaning period emerges as the potentially ideal time for microbial interventions.

## The weaning period as the ideal time for microbiome interventions

### Microbiome ecological framework

Macroecological theory is a powerful lens for understanding microbiome development and the challenges of implementing microbial interventions (Table 2). The adult intestinal microbiome is a climax community where the available microbial niches are occupied.^213^ Probiotics typically do not colonize the gut to detectable levels in adults because it is difficult for exogenous organisms to compete for limited space and resources against community members that are already established.^214^ Lower density and diversity communities, like the infant microbiome, are more conducive to the introduction of new organisms because there is less competition for resources. Fecal-microbiota transplant (FMT) is a microbial intervention used to treat recurrent *C. difficile* infection (rCDI) that likely works under the same principle. FMT entails seeding a recipient with fecal microbes from a healthy donor.^215^ Before FMT, the recipient typically receives antibiotic treatment for rCDI and often a bowel purge to deplete their microbiota. These pre-treatment interventions create available niches for the new community of transplanted microbes to colonize, which is critical for the successful engraftment of the donor community.

**Table 2.** Advantages and challenges of microbiome-directed therapies.

Microbiome-targeted Therapeutic	Advantages	Challenges
Fecal Microbiota Transfer	Complete community with intact network of cooperative relationships Typically preceded by antibiotics treatment which liberates space and resources for new microbes	Must recruit and screen potential “healthy donors” Variability between donors Undefined community Involves stringent manufacturing, shipping, and storage conditions Typically administered in a hospital setting Risk of infection/adverse events
Probiotics	Defined single strain or consortia Typically microbes with known benefits to the host Commercially available	Engraftment into a complex, mature community is rare Often involves stringent manufacturing, shipping, and storage conditions May require specific dietary substrates in order to generate beneficial metabolites Risk of infection/adverse events
Prebiotics	Less stringent manufacturing, shipping, and storage conditions Commercially available Can leverage foods that are already part of the target population’s diet No risk of infection	Requires the presence of microbes that can utilize the dietary substrate Often does not target a specific microbe of interest Sustained effects require continuous dosing
Synbiotics	Defined single strain paired with specific dietary substrate to produce known metabolites Promotes engraftment as long as the nutrient is present (“tunable”) Commercially available	Often involves stringent manufacturing, shipping, and storage conditions for the microbial strain Limited known instances of beneficial microbes that have a significant growth advantage in the presence of a specific nutrient Risk of infection/adverse events
Postbiotics	Less stringent shipping and storage conditions Contains the specific metabolite or microbially-derived molecule that mediates host benefits No risk of infection	Sustained effects require continuous dosing
Parabiotics	Less stringent shipping and storage conditions No risk of infection	Some probiotic strains must be alive to confer benefits to the host Sustained effects require continuous dosing

Even when niches are available, a new organism might require help from other organisms to successfully integrate into a community. Mature ecosystems include complex networks of symbiotic and commensal relationships that increase fitness by allowing organisms to access additional resources.^104,216^ An example of this in the gut is cross-feeding where “microbe A” produces metabolic byproducts that can be used as nutrients by “microbe B”.^217^ The gut commensal *Anaerostipes caccae* cannot grow *in vitro* on starch as a sole carbon source but can grow in co-culture with *Bifidobacterium adolescentis* because *B. adolescentis* can metabolize starch to produce lactate which is then utilized by *A. caccae* to produce butyrate.^218^ In the context of the post-weaning lower gastrointestinal tract, where complex carbohydrates like starch are the predominant microbially available carbon source, it is unlikely that *A. caccae* would be able to engraft without the presence of another microbe, like *B. adolescentis*, to metabolize those complex carbohydrates into a usable carbon source, like lactate. This is just one example of the many cross-feeding relationships present in the mammalian intestinal microbiome (reviewed extensively by Culp & Goodman).^219^ Coyte et al.^130^ used ecological network theory to model microbiome assembly and found that the probability of reaching a climax community was very low when individual species entered the environment one at a time, as the absence of a critical cooperative partner could result in the collapse of the entire community. Adjusting their model to allow multiple species to enter the environment simultaneously resulted in a higher probability of reaching a climax community by increasing the likelihood that cooperative partners were both present. Again, this finding is supported by the clinical success of the only live microbial therapeutics that are FDA approved in the United States, VOWST and Rebyota.^8^ Because both of these drugs treat rCDI with microbial consortia isolated from healthy communities, stable engraftment is supported by preexisting cooperative networks. It is thought that a similar process occurs at birth when a baby is simultaneously exposed to their mother’s entire vaginal or skin microbiome.^154^ These theoretical and experimental findings argue that introducing communities of microbes, rather than single strain, is a more effective strategy for manipulating the intestinal microbiome.

Another way to increase the fitness of a desired microbe and foster stable colonization of the gut is to use dietary supplements called prebiotics. Prebiotic supplements are usually complex carbohydrates that cannot be metabolized by the host and thus add microbially-available nutrients to the gut environment to promote the growth of beneficial microbes. In their ecological network model, Coyte et al. ^130^ found that increasing nutrients early in community development promotes assembly by decreasing reliance on primary colonizers to liberate nutrients for secondary colonizers. Prebiotic supplements can have specific and predictable effects on the community if there is a microbe present that is uniquely equipped to compete for that nutrient source, such as in the case of *Bifidobacterium* species and HMOs. In a study where adults consumed HMO concentrate, the *Bifidobacterium* species in participants’ guts detected before the start of the study expanded in a dose-dependent manner to the HMO supplementation.^220^ The effects of prebiotic supplements are more robust when combined with a live microbe that competes well for that nutrient, an intervention sometimes called “synbiotics”. Another study gave adults a combination of HMOs and live *B. infantis* and saw that as long as participants kept consuming HMOs, *B. infantis* persisted in their gut, even once they stopped taking the live microbe, resulting in what they called “tunable engraftment”.^221^ This is very encouraging given that a recent randomized control trial found that a strain of *B. infantis* given to breastfeeding infants lead to a dampening of inflammatory T cell subsets and cytokines.^210^ These studies provide a theoretical framework and real-world evidence for modulating the microbiome through weaning-associated nutrients and commensal microbes.

Dietary interventions also avoid the logistical challenges of culturing and delivering live microbes. Most beneficial microbes found in a healthy adult microbiome are strict anaerobes, have complex growth requirements, or have never been successfully grown *in vitro*.^222,223^ FMT circumvents the issue of cultivability by extracting microbes directly from feces, while most commercially available probiotics, such as *Lactobacillus* species, are aerotolerant and less fastidious. Even after initial isolation and production, many live microbial therapeutics, including FMT, must be kept frozen or refrigerated throughout the supply chain to maintain viability and effectiveness.^224^ This renders the implementation of probiotic interventions particularly difficult in low-resource settings. Gehrig et al.^225^ tackled these challenges while designing a microbiome-directed intervention to treat malnourished children in Bangladesh. Malnutrition is associated with a dysbiotic microbiome that often does not recover after refeeding.^226^ A persistently dysbiotic microbiome is linked to metabolic dysregulation and poorer long-term growth outcomes in children treated for malnutrition.^226,227^ Gehrig et al. ^225^ used humanized murine and porcine models of malnutrition-associated dysbiosis to screen complementary foods typical of their study population. They identified a combination of foods that promote healthy microbiome maturation and recovery from metabolic dysfunction and used those ingredients to generate a palatable formulation that successfully treated malnourished children. The success of this intervention demonstrates the feasibility of using familiar and accessible foods to develop diet-based interventions that promote healthy microbiome development.

Another growing area of microbiome-directed therapeutics that avoids the challenges of live microbial therapeutics is the use of microbially-derived molecules, sometimes called “postbiotics.” For the purposes of this review, postbiotics refer to metabolites or other molecules that are secreted by live microbes or released upon microbial lysis.^228^ For example, short-chain fatty acids (SCFAs) produced by the microbial metabolism of complex carbohydrates promote induction of regulatory T cells (Tregs), regulate intestinal IgA, and activate innate lymphoid cells, among other functions.^229^ In some studies, short-chain fatty acids can be given as a dietary supplement to induce these immunomodulatory effects, indicating that the microbes that typically produce them *in vivo* are not required.^230^ Another group of potential postbiotics of interest are secondary bile acids which are produced by metabolism of host-derived primary bile acids by gut microbes. There is growing evidence that these microbial bile acid metabolites modulate the immune system and prevent autoimmunity.^231^ A similar class of therapeutics, called “parabiotics”, involve the use of non-viable whole microbes. For example, several clinical trials have demonstrated that, for multiple strains of probiotics, live and heat-killed cells confer the same health benefits.^228^ Both postbiotics and parabiotics provide similar feasibility benefits to prebiotics, because they can forgo the stringent manufacturing, shipping, and storage conditions needed to keep live cultures viable. Although, they require more specialized manufacturing processes than using readily available, culture-specific prebiotic foods. Post- and parabiotics can also provide more targeted effects than prebiotics because they may act downstream of the microbiome and therefore do not require the presence of a specific set of microbes to modulate the host.^228^ Lastly, they do not pose the same risks as live microbial therapeutics, including the potential for probiotics to translocate out of the gut and cause systemic inflammation.^228^ Thus, postbiotics and parabiotics represent a therapeutic option that minimizes feasibility challenges while still providing a targeted intervention.

In summary, microbial ecology principles predict that the introduction of new species to an ecosystem is most likely to succeed when the preexisting community has low density and diversity so there are available niches; when other new species arrive at the same time to establish cooperative relationships; and when there are additional resources that the new species can effectively utilize. The early-life gastrointestinal tract of weaning mammals meets these criteria and provides the ideal conditions for introducing microbial therapeutics (Table 3).

**Table 3.** The weaning-associated intestinal microbiome exhibits critical features that allow new microbes to enter the community and stably colonize.

Features of receptive and expanding microbial ecosystems	Features of the intestinal microbiome at weaning	Features of the intestinal microbiome in adulthood
Low density	✓	-
Low diversity	✓	-
New nutritional resources	✓	-
Open/changing mucosal niches	✓	-
Exposures to new microbes	✓	✓
Receptive host	✓	-

### Physiological changes in the host facilitate the microbiome weaning transition

Studies in animal models support the hypothesis that weaning is an opportune time for microbial interventions. The 10 days following weaning are the most dynamic period during microbiome ontogeny in mice.^232^ In mice, introducing a donor microbial community at weaning leads to better long-term colonization than in adult mice.^233^ Interestingly, alpha diversity was similar between experimental groups prior to the introduction of the donor community. This suggests that there are other characteristics of the microbiota and/or host physiology at weaning that facilitate engraftment of novel microbes. For instance, the transitory nature of the weaning microbiome may lead to instability in the community that provides an opportunity for novel microbes to establish a niche.

Evidence from non-human primates also suggests that weaning is an optimal period for probiotic microbe colonization. Like human infants, young chimpanzees begin consuming solid food at around six months of age but have an extended complementary feeding period, lasting up to four years.^234^ The alpha diversity of chimpanzee infants <2 years old is higher than that of adults.^235^ This finding in chimpanzees suggests that the complementary feeding period can support a more diverse range of microbes than after the cessation of breastmilk.

This is reflected by the non-linear dynamics of microbiome beta diversity in human infants prior to and during weaning. At birth, intestinal microbiome beta diversity is high between infants, which decreases up to the point of solid food introduction when it rapidly rises.^236^ This sharp increase in beta diversity peaks at ~2 months after the introduction of solid foods, at which point beta diversity gradually decreases again. The complementary feeding period has the additional benefit of allowing early-life-associated microbes to persist during the acquisition of adult microbes. This is particularly important since the most extensively studied probiotics are early-life associated, e.g., *Bifidobacterium, Lactobacillus*, *Streptococcus*, and *Enterococcus* species and *Akkermansia muciniphila*. ^237,238^ The pattern of fluctuating beta diversity at weaning and the pre-weaning association of many probiotic species highlights the weaning transition as a period that provides a unique opportunity to introduce new microbes.

In addition to the changing nutrient landscape, several host-intrinsic factors support the colonization of novel microbes during weaning. Reduced breastmilk intake during weaning decreases the exposure to the maternal immune factors that modulate the microbiome. Key modulators include maternal immunoglobulins (mainly sIgA) and proteins with anti-microbial activity (lactoferrin, lysozyme, complement). sIgA has pleiotropic functions including aggregating microbes to enhance clearance, modulating microbial gene expression, and promoting colonization of other specific microbes by facilitating residence within the mucosa.^209^ While the concentration of maternal sIgA in the infant gut declines during weaning, endogenous sIgA levels do not reach adult levels until 2 years of age.^239^ This physiologic nadir in sIgA is an important ecologic perturbation that may allow the introduction of beneficial microbes to intestinal niches during weaning. As maternal factors are lost at weaning, cell intrinsic factors trigger maturation of the intestinal epithelium. Studies in mice have shown that this weaning-associated transcriptional remodeling occurs with the same kinetics, even when enterocytes are removed from the intestinal microenvironment.^240,241^ Genes that are upregulated at weaning include antimicrobial peptides, lysozyme, and carbohydrate metabolizing enzymes.^241^ These hallmarks of a mature epithelium likely impact which microbes persist close to the epithelium.

Important structural and functional changes in the mucosa during postnatal development support the weaning transition, including changes in the mucus layer that enhance gut barrier function. Mucins, the primary component of the intestinal mucus layer, are a family of high molecular weight, highly glycosylated proteins that are classified into sulfated and non-sulfated subtypes based on the terminal moiety present on the glycan.^242^ Non-sulfated mucins either have a sialic acid or fucose terminal sugar, further classifying mucins as either acidic or neutral, respectively. Human fetal mucins contain more neutral glycans than the more sialylated adult mucins.^243^ In adults, mucin glycosylation forms a gradient of increased sialylation over fucosylation of mucins from the ileum to the distant colon which is not seen in fetuses. It is not known exactly when this shift to an adult glycosylation pattern occurs in human infants. In mice, a shift from predominately sialylated to fucosylated mucins occurs during weaning.^244^ This transition alters the nutrient availability and adherence of mucosa-associated microbes.^245,246^ Germ-free mouse studies determined that increased mucin fucosylation is mediated by bacterial colonization, and that fucosylation occurs in the large intestine but not the small intestine.^247^ This selective fucosylation of colonic mucin mirrors the pattern of mucin glycosylation seen in adult humans. Fucosyltransferase 2 (Fut2) activity in human infants alters the intestinal microbiome, with higher secretion of Fut2 correlating to higher alpha diversity and distinct microbial communities.^248^ This finding suggests that alterations in mucosal glycosylation during weaning can shape microbiome community composition during this period. Similarly, alterations in the mucosa by changing sIgA levels and mucin architecture are likely important variables that regulate probiotic colonization and function. Indeed, many of the most studied probiotic species can either consume mucin glycans or adhere to mucin or the epithelial layer.^249–251^ Host-microbe interactions at the mucosal surface are likely important for the beneficial functions of these microbes. Coupled with the fact that many probiotic species are early-life associated, the changes in the host gut environment during the weaning transition makes it an ideal time for the introduction of beneficial microbes.

Caution is warranted when translating discoveries from neonatal rodent models to human infants. Neonatal rodents are less developed at birth than human infants; 14-day-old pups resemble a ~ 24-week-old fetus in intestinal development.^252^ Several features that likely influence host-microbe interactions change during the weaning period in rodents but occur during the fetal period for humans, including crypt formation,^253^ Paneth cell development,^254^ anti-microbial peptide generation,^255^ and mucin production.^256,257^

### Window of opportunity for microbial therapeutics

Early life is a critical time for developing homeostatic immune responses because the immune system is more susceptible to imprinting by microbiota and is skewed toward a tolerogenic state.^258^ Weaning-associated immunological changes include the induction of peripheral Tregs, mucosal-associated invariant T (MAIT) cells, invariant natural killer T (iNKT) cells, endogenous IgA production, and mucus secretion, among others^259^ (Figure 1). Epidemiological studies in humans have shown that formula feeding or treatment with antibiotics in early life is associated with an increased risk of asthma and allergy.^260–262^ Therefore, disruption of host-commensal interactions in early life can have negative long-term health consequences. Conversely, children who are raised in rural environments,^263,264^ consume unpasteurized milk,^265,266^ or grow up around pets^267^ have a lower risk of autoimmune and inflammatory disease, likely due to increased exposure to environmental microbes. These early-life risk factors highlight the need for microbial therapeutics targeting this critical stage in development.

Literature on the benefits of introducing microbes during weaning rather than post-weaning is limited. Germ-free mice provide the clearest evidence of the importance of microbial intervention during weaning. Colonizing germ-free mice with microbiota before weaning reduced the severity of dextran sodium sulfate (DSS) mediated colitis,^11^ while introducing microbiota to post-weaning germ-free mice did not ameliorate DSS colitis. Knoop et al. ^10^ demonstrated that simply changing the timing of microbial antigen exposure from weaning to post-weaning shifts commensal-specific immune responses from tolerogenic to inflammatory. The timing of microbial antigen exposure is controlled by the reduction of milk-derived epidermal growth factor (EGF) during weaning, triggering the opening of goblet-cell-associated passages in the intestinal epithelium.^10,11^ Cahenzli et al. ^212^ demonstrated that introducing microbiota during weaning was sufficient to restrain the development of abnormally high serum IgE levels in mice born germ-free, while colonization in adult mice did not affect IgE levels. Probiotic administration during the pre-weaning period significantly reduced lung inflammation compared to probiotic-treated adult mice in an asthma model.^211^ We recently reported that colonizing germ-free non-obese diabetic (NOD) mice with a defined consortium of early-life microbes before weaning reduces diabetes incidence.^268^ However, colonization with the same consortium in adult germ-free NOD mice had no impact on diabetes incidence. Tregs induced by early-life microbes are thought to be important mediators of immune tolerance in these models. Additionally, Olsak et al. ^269^ found that microbial colonization of neonatal germ-free mice, but not adults, reduced inflammatory responses associated with asthma and colitis. This study determined that microbial exposure in early life reduced invariant natural killer cell populations to levels seen in conventional mice by lowering C-X-C motif chemokine ligand 16 (*Cxcl16*) expression through epigenetic alterations in methylation of the *Cxcl16* gene. Why microbial exposure must occur during early life to regulate iNKT cell populations is unknown. Also, the specific microbes required for long-term immune system modulation is an open question in the field. Overall, the beneficial immunomodulatory properties of some potential probiotics may be limited to the early-life period. More investigation is needed into differential responses to probiotics at weaning versus later in life.

## Conclusions and next steps

In this review, we summarized the changes in the available nutrients during the weaning transition from breastmilk and formula to solid foods, the impact of complementary feeding on intestinal microbial communities, and the physiological changes in the host that may facilitate the introduction of new microbes at weaning. We propose that the low-density, low-diversity state of the pre-weaning microbiome coupled with developmental changes in the mammalian gut and immune systems during the weaning transition make this developmental stage an ideal time for microbial interventions to provide lasting impacts on health. As most microbiome studies are conducted on fecal samples, the impacts of weaning-induced shifts in microbial populations in the small intestine and mucosal surfaces remain a critical gap in knowledge. A better understanding of the weaning dynamics will provide an important foundation for designing more effective immunomodulatory probiotics (**Box 1**).

**Box 1.** Areas for future research and open questions

Intestinal Biogeography
a. Tools to study the small intestinal microbiome
i. Non-invasive measurements (breath metabolites)
ii. Minimally invasive measurements (swallowed capsules)
b. Standardized collection of mucus-associated microbiota samples
c. How does the mucosal microbiome change during weaning?
(2) Body sites outside of the gut
a. Influence of gut microbes and metabolites on distal body sites
b. Weaning-associated changes in the microbiota at other barrier sites
c. What is the relationship between microbiome changes at weaning and neuro-immune development?
(3) Microbiota dynamics across lifespan
a. Analytic tools to synthesize longitudinal, multi-omics datasets and identify significant microbiome and immune trajectories over lifespan
b. How do different windows of microbiome and immune system development (prenatal, pre-weaning, and post-weaning) relate to each other?
(4) From association to causation and translation
a. Modeling microbiome and metabolic dynamics using both absolute and relative abundance measurements
b. Humanized gnotobiotic models of the early-life microbiome
c. Equitable microbiome studies across diverse populations
d. Can the beneficial features of the weaning microbiome be replicated in adulthood to enhance efficacy of microbial therapeutics?
